# Severe bacterial skin infections^☆^^☆☆^

**DOI:** 10.1016/j.abd.2020.04.003

**Published:** 2020-05-16

**Authors:** Sílvio Alencar Marques, Luciana Patrícia Fernandes Abbade

**Affiliations:** Faculdade de Medicina, Universidade Estadual Paulista, Botucatu, SP, Brazil

**Keywords:** Bacterial infections, Ecthyma, Fasciitis, necrotizing, Fournier gangrene, Furunculosis, Methicillin-resistant *Staphylococcus aureus*

## Abstract

The severe bacterial diseases discussed herein are those that present dermatological lesions as their initial manifestations, for which the dermatologist is often called upon to give an opinion or is even the first to examine the patient. This review focuses on those that evolve with skin necrosis during their natural history, that is, necrotizing fasciitis, Fournier gangrene, and ecthyma gangrenosum. Notice that the more descriptive terminology was adopted; each disease was individualized, rather than being referred by the generic term “necrotizing soft tissue infections”. Due to their relevance and increasing frequency, infections by methicillin-resistant *Staphylococcus aureus* (MRSA) were also included, more specifically abscesses, furuncle, and carbuncle, and their potential etiologies by MRSA. This article focuses on the epidemiology, clinical dermatological manifestations, methods of diagnosis, and treatment of each of the diseases mentioned.

## Introduction

This article aims to review severe infectious conditions caused by bacteria that, either due to skin involvement as a primary manifestation, or due to a skin manifestation that indicates severe systemic involvement, must be considered as mandatory knowledge for dermatologists, regardless of their main area of expertise. Currently, several authors, particularly in the field of infectious diseases and surgery, prefer to use the term necrotizing soft tissue infections (NSTIs), which includes the various infections that evolve to skin necrosis, such as necrotizing fasciitis (NF), Fournier gangrene, Meleney's synergistic gangrene (post-operative), gas gangrene, necrotizing cellulitis, and myonecrosis.^1^, ^2^, ^3^ However, for greater clarity and considering the prevalence of these conditions, the study adopts the classic terminology of NF and Fournier gangrene, and includes ecthyma gangrenosum (EG) and cutaneous infections caused by methicillin-resistant *Staphylococcus aureus* (MRSA), with emphasis on furunculosis and abscesses. The review of the host's defense mechanisms, bacterial resistance to antibiotics, virulence, and microbiome is beyond the scope of this article; therefore, only the essential mechanisms to understand the etiology and physiopathogenesis of the disease under discussion were included.

## Necrotizing fasciitis

NF is an unusual, acute, fast-progressing infectious process that evolves with superficial and even deep muscle fascia necrosis of the subcutaneous tissue, dermis, and epidermis, and can progress to sepsis, shock, and death in up to 40% of cases.^4^, ^5^ Under the name “hospital gangrene,” this condition was described in the 19th century, with the publication, in 1871, of 2642 cases observed by surgeon Joseph Jones of the Confederate army during the American civil war, as mentioned by Faraklas et al.^3^ In 1952, American surgeon B. Wilson coined the term necrotizing fasciitis, currently in universal use, to designate the infectious processes that progress through the plane of the superficial muscular fascia and that evolve to necrosis of the subcutaneous tissues to the epidermis, and even to the deep fascia and muscular plane.^6^

In the absolute majority of cases, NF starts by inoculation of the pathogen or pathogens through injury to the skin resulting from trauma, perforating injuries, human or animal bites, insect bites, small procedures, catheter insertion, injection of medication or illicit drugs, and post-varicella complications, among others, including contusion without loss of continuity in the skin.^7^, ^8^, ^9^, ^10^, ^11^ The most common topographic location of NF is the lower limbs, but involvement of the upper limbs (including the hands), head, face, neck, and trunk are not exceptional.^11^, ^12^, ^13^, ^14^, ^15^ The condition presents no predilection for sex or age, being observed in children and young people, but it is more frequent in adults and elderlyy. The main predisposing factors are age, diabetes mellitus, obesity, alcoholism, malnutrition, immunosuppression, chronic kidney or liver disease, peripheral vascular disease, and chronic use of illicit drugs, but it may even occur in seemingly healthy individuals.^10^, ^11^, ^12^ Recently, NF has been reported in association with the use of targeted therapies, such as tyrosine kinase inhibitors; however, the cause-effect relationship in the reported cases is difficult to define, given the presence of several possible cofactors in these patients.^16^ From an etiopathogenic standpoint, the condition progresses rapidly due to the action of endo- and exotoxins produced by the infecting bacterial species, evolving to obliterating endarteritis, local microcirculatory thrombosis, and necrosis.^4^, ^7^, ^12^ If the condition is left untreated, is treated late, or is only treated clinically, it can evolve to toxemia, sepsis, disseminated intravascular coagulation, multiple organ failure, and death.^7^, ^11^, ^12^

A widely used NF classification designates as type I the disease caused by a synergistic association of aerobic or anaerobic bacteria; therefore, polymicrobial necrotizing fasciitis, which includes Group A *Streptococcus*, *Staphylococcus aureus* or other species of *Staphylococci*, *Escherichia coli*, *Klebsiella* spp., and, less often, other Gram-negative bacteria.^4^, ^7^, ^17^ The estimated frequency of this type I ranges from 70% to 80%.^4^ Type II NF, which is monomicrobial, is also termed “streptococcal gangrenous cellulitis.” It is caused by Group A beta-hemolytic streptococci (also termed flesh-eating bacteria), and its frequency is estimated between 20% and 30% of NF cases.^7^, ^18^, ^19^ The most frequent species of *Streptococcus* is *Streptococcus pyogenes* serotypes M1 and M3, which have the ability to modify the host's phagocytic defense response, produce hemolysins and exotoxins with superantigen properties and, consequently, induce excessive proliferation of T lymphocytes and release of pro-inflammatory cytokines.^7^, ^20^ These act as triggers for the streptococcal toxic shock syndrome, which can occur in up to 14% of severe streptococcal infections and are associated with high mortality.^7^, ^19^ It is important to remember that septic shock is a syndromic condition triggered by a severe infectious condition, either by Gram-positive or Gram-negative bacteria, and that results in the release of pro-inflammatory mediators, including TNF-alpha, produced by monocytes, neutrophils, and macrophages, which induce vasodilation, extravasation of plasma from the vessels, cardiac malfunction, immunosuppression, and multiple organ failure.^7^, ^20^ It is not uncommon for *Staphylococcus aureus* or other species of staphylococci to be associated with streptococcal infection, potentiating its deleterious effects.^21^

**Figure 1:**
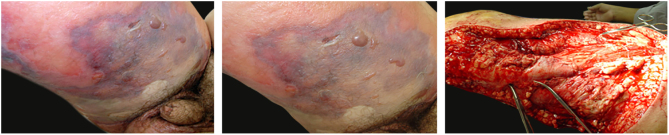
Necrotizing fasciitis. (A) Overview showing the extent of damage and different clinical stages. (B) Detail of the pale, erythematous-violaceous, painful devitalized area. Blister with hemorrhagic content and marginal erythema in the still viable area. (C) Demonstration of the necessary widening of surgical debridement.

**Figure 2:**
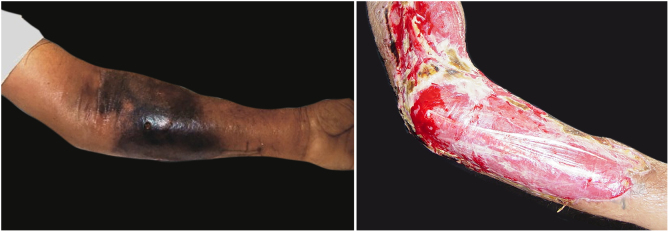
Necrotizing fasciitis after manipulation of furunculoid myiasis. (A) Presence of edema, mild erythema, and extensive area of necrosis. (B) Debridement product in which necrosis foci are still observed.

**Figure 3:**
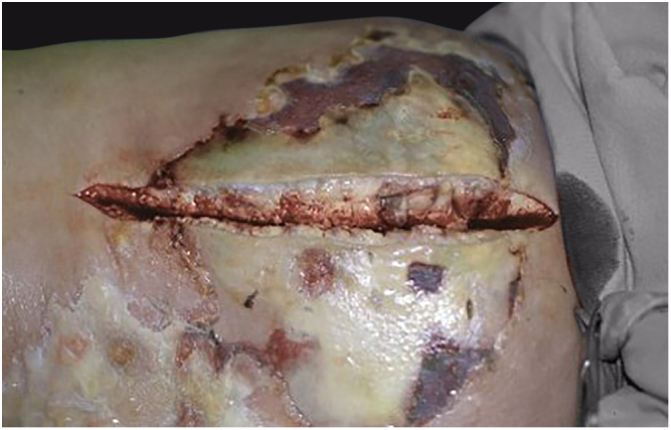
Necrotizing fasciitis of the medial aspect of the thigh with a consolidated, devitalized lesion; upon incision, almost no bleeding was observed due to thrombosis of the perforating vessels from the fascia to the epidermis.

The initial clinical manifestation may be subtle and discreet, with erythema, edema, and local heat suggesting a picture of erysipela or cellulitis, which delays the diagnostic suspicion related to severity. However, pain disproportionate to the apparent benignity of the condition is noteworthy. As a rule, there is a rapid evolution (in 24–72 h) to an erythematous-violaceous condition; the limits of the lesion are lost, hemorrhagic vesicles are observed, and infiltration is identified by firm tissue induration, edema, and pain beyond the noticeable limits of the erythema (Figure 1, Figure 2). Subsequently, areas of pallor are observed; the pain disappears or is markedly reduced as a result of the necrosis of the local nerve branches. In addition to these dermatological signs, general signs of fever, lethargy, mental confusion, toxemia and, potentially, sepsis and shock are observed in various degrees. As a consequence of thromboembolism of the local/regional microcirculation, there is practically no bleeding during the biopsy procedure or local exploratory surgical incision (Fig. 3). Depending on the duration of the clinical history, signs of cutaneous emphysema and a foul-smelling discharge may be observed during the procedure, indicating tissue necrosis. Computed tomography (CT) or magnetic resonance imaging (MRI) are useful to demonstrate the presence of subcutaneous edema, fascia thickening, collection of purulent material, and even the presence of gas, an evidence of severity that distinguishes NF from severe cellulitis.^22^, ^23^, ^24^ Deep incisional biopsy, down to the fascia, or frozen cuts for quick analysis, may also be useful. However, requesting and waiting for such procedures require time, which can be crucial for these cases. If the diagnosis is not established or if appropriate interventions are not instituted, signs of sepsis manifest themselves between the fourth and sixth day of infection, and may be anticipated or postponed. Therefore, diagnostic suspicion based on the history and clinical and dermatological examination must be raised as early as possible, prescribing broad antibiotic coverage and indicating surgical debridement.

The Laboratory Risk Indicator for Necrotizing Fasciitis (LRINEC), proposed by Wong et al. in 2004, is a scoring system that can be a useful diagnostic aid, despite the lack of consensus on its specificity. The LRINEC uses data from c-reative protein (CRP), leukogram, hemoglobin, serum sodium, creatinine, and glycemia collected when the patient is first seen or when NF is first suspected, which are graded according to the intensity of the detected laboratory alteration. A final score of 6 would be equivalent to an intermediate risk of NF and scores equal to or greater than 8 are indicative of a high risk of NF (Table 1).^25^, ^26^ In addition to this laboratory data, it is important to gather the information obtained from manipulation of the lesion by a surgeon in order to preliminarily incise and expose the tissue, and to evaluate the presence of gross necrosis of the fascia and whether the fascia can be easily detached from its superior planes. In case of suspicion, biopsy and culture of tissue fragments should be performed and blood culture should be collected more than once.

**Table 1:** Laboratory data indicative of risk for the diagnosis of necrotizing fasciitis (Laboratory Risk Indicator for Necrotizing Fasciitis [LRINEC]).

Parameters	Values	Score (%)
Hb (g/dL)	>13.5	0
	11–13.5	1
	<11.0	2
Leukocytes (10^9^/L)	<15	0
	15–25	1
	>25	2
Sodium (mmoL/L)	<135	2
Creatinine (moL/mL)	>1.41	2
Glucose	>100	1
C-reactive protein	>15	4

The sum of scores < 5, ≤50% risk (low risk).

Between 6 and 7 = intermediate risk; >8 = 75% risk (high risk).

The differential diagnosis includes infectious conditions of the skin, subcutaneous tissue and muscle, including erysipelas and severe, bullous cellulitis, abscesses, infected hematoma, pyoderma gangrenosum, pyomyositis, gas gangrene, and postoperative or post-procedure infection. The semiological knowledge, and the peculiarities of dermatological and clinical history aided by laboratory data, shown in table 1, raise diagnostic suspicion and allow clinical diagnosis.

Antibiotic coverage should be instituted immediately in the face of suspicion; it is not necessary to wait for a final diagnosis. The antibiotic therapy should be able to cover, albeit empirically, polymicrobial NF, acting against Gram-positive, Gram-negative, and anaerobic bacteria. An attempted analysis through systematic review, using the Cochrane methodology, did not result in definitive information regarding antibiotic therapy in necrotizing infections affecting soft tissues.^27^ In summary, those authors concluded that: “There is no empirical antimicrobial therapy validated by clinical trial. … topics (items) should be considered, among which the antimicrobial strategy in patients with or without comorbidities and in patients with a risk factor for MRSA infection.”^27^

Based on literature data and the personal experience of the authors, the use of one of the following schemes is suggested: (1) Piperacillin sodium + tazobactan sodium (active against Gram-positive, even if producers of β-lactamases, active against Gram-negative, not active against MRSA), + vancomycin (active against MRSA) + clindamycin (active against Gram-positive aerobic and anaerobic bacteria).^7^, ^9^, ^11^, ^17^, ^18^, ^19^, ^21^ (2) Imipenem/meropenem (of the carbapenem class; active against Gram-positive, even if producers of β-lactamases, active against Gram-negative, not active against MRSA) + vancomycin + clindamycin. (3) Cefepime (fourth-generation cephalosporin; active against Gram-positive, even if producers of β-lactamases, active against Gram-negative, not active against MRSA), + vancomycin + clindamycin. The results obtained from culture/blood culture and antibiogram may lead to adjustments in the proposals above, particularly in the assessment of the need to associate an antibiotic of the aminoglycoside class to the scheme. If necessary or convenient, clindamycin can be replaced by metronidazole 1 g every 12 h, intravenously. It is important to note that several of the drugs listed above require correction for creatinine clearance when indicated; in children below 40 kg or neonates, the dose must be adjusted.

**Figure 4:**
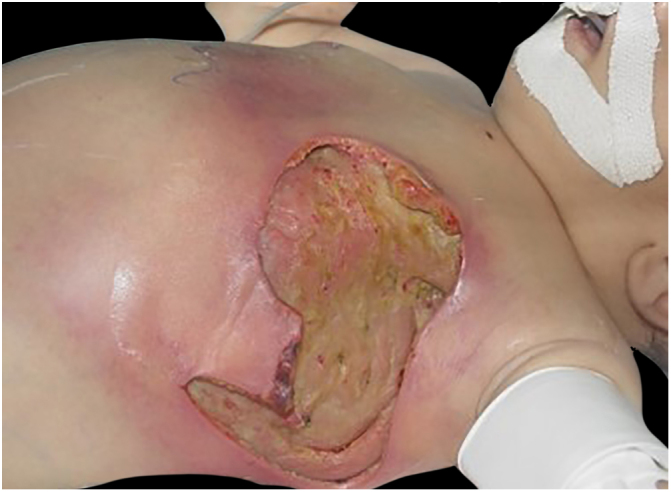
Necrotizing fasciitis in a nursing infant after an attempt to squeeze dripping milk from the nipple. The area was debrided until the muscle plane, with removal of the fascia. Presence of infectious activity an area of the chest.

Associated with antibacterial treatment, surgical debridement is also an essential emergency procedure (Figure 1, Figure 2). It is not uncommon for the dermatologist to have to convince the emergency surgeons of the need for the procedure. The debridement must be wide, excising all necrotic subcutaneous tissue including the fascia, and going beyond the gross necrosis as a safety margin (Fig. 4).^1^, ^2^, ^7^, ^11^, ^17^ It is necessary to assess whether there is associated myonecrosis and whether the involved limb must be amputated. The surgical wound must remain open and be reassessed in 24 h; if necessary, the excision must be extended, as the infectious process and thrombosis of the local microcirculation often progress despite antibiotic therapy.

As an adjunct treatment to broad-spectrum antibiotic therapy and surgical debridement, there are reports of the use of intravenous immunoglobulin (IVIG), hyperbaric oxygen therapy, and negative pressure therapy (NPT). IVIG is mentioned as a treatment in cases associated with septic shock, due to the possibility of neutralizing the formation of superantigens.^28^ However, a retrospective cohort study of 164 patients concluded that IVIG had no apparent impact on mortality or length of hospitalization beyond that achieved with debridement and antibiotics.^29^ Regarding hyperbaric oxygen therapy, a Cochrane systematic review failed to demonstrate relevant clinical evidence to support or refute the effectiveness of this therapy in NF.^30^ The literature features many articles (primarily case reports and series) on the use of NPT, a method used to accelerate healing; however, randomized clinical trials to ensure its validity are lacking.^31^, ^32^ It is important to emphasize that supportive and intensive care measures must be available and used as indicated. With the patient's recovery, the surgical wound can be repaired by local grafting when the bottom is clean and tissue granulation is observed, or by healing by second intention.^17^ Esthetic, functional, or social sequelae deserve equal effort to be reduced or solved in the best possible way.

## Fournier gangrene

In 1883, the French dermatologist Jean-Alfred Fournier (1832–1914), head of the Department of Dermatology and Syphilography at the Hôpital Saint Louis de Paris, reported an infection and rapid evolution to necrosis of the perineum and scrotum in five patients; he termed this evolution *gangrène foudroyante de la verge*, *i.e.*, rapid gangrene of the penis.^33^, ^34^ Currently, this condition is recognized as a variant of necrotizing fasciitis with an initial and specific location in the perineum, genitalia, or perianal region, and is now known as Fournier gangrene.^34^ It is an uncommon disease, whose mortality rates range from 7.5% to 22.5% of cases in different series.^34^, ^35^, ^36^ It can be confined to the scrotum or extend toward the perineum, penis, pubis, and abdominal wall. Although more frequent in males, who account for 52% to 100% of cases in different series, it can also be observed in females.^34^, ^35^, ^36^ The age group most affected is that above 50 years of age, but cases in children and adolescents, although very rare, have been described.^34^, ^35^, ^36^ In a review study of 40 cases diagnosed in Brazil, the authors identified diabetes mellitus as the main comorbidity, present in 70% of patients, followed by systemic arterial hypertension (35%), heart disease (15%), and dyslipidemia and obesity in 7.5%.^37^ Alcohol abuse and malnutrition are also mentioned as predisposing factors. The triggering factors are varied; prior infection of the urinary tract, perianal infection, surgical manipulation (including postectomy), penile prosthesis, genital trauma (including penis-enlargement fillings), and scrotal trauma are listed as the most important and frequent.^34^, ^35^, ^36^, ^37^, ^38^, ^39^ In women, traumas, microtraumas related to hair removal, episiotomy, and infection of the vulvar region and perineum are mentioned.^34^, ^35^

The etiology is polymicrobial in most cases, ranging from 54% to 80% in different series. The most common infectious agent is *Escherichia coli*, but bacteria of the genera *Streptococcus*, *Bacteroides*, *Enterobacter*, *Staphylococcus*, including MRSA, *Enterococcus*, *Pseudomonas*, *Corynebacterium*, *Klebsiella*, or even *Candida albicans* are also common. Such agents can act alone or in association.^35^, ^40^, ^41^, ^42^

Fournier gangrene progresses through the superficial and deep planes of the urogenital and anogenital fascia.^34^ The sequence of events mirrors that of classic cutaneous NF: infection, vascular occlusion, infarction, and tissue necrosis. The condition progresses very quickly in males, as Colles’ fascia of the perineum, Dartos’ fascia of the penis and scrotum, and Scarpa's fascia of the anterior abdominal wall form a continuum, allowing the infection to progress through these planes.^7^, ^34^

**Figure 5:**
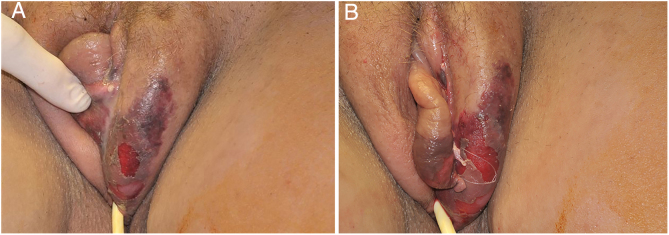
Fournier gangrene on the vulva after hair removal. Presence of edema, erythema, and signs of necrosis.

The initial condition is erythema and edema with increased volume and, as in classical NF, is accompanied by pain that is disproportional to the clinical appearance.^34^, ^35^, ^37^ The sequence of dermatological signs can be described as edema, swelling, poorly defined erythema, violaceous erythema, and finally pallor and cutaneous necrosis, also identified in cases of vulvar lesions (Fig. 5).

Clinical evaluation and diagnostic suspicion must be raised as early as possible and the intervention must not wait for histological or microbiological confirmation. Although the diagnostic referral can be dermatological, the intervention is urological, gynecological, and surgical, aiming to remove all devitalized tissue, and combined with antibiotic coverage targeted at the aforementioned pathogens.^35^, ^40^, ^41^, ^42^ The antibiotic prescription cannot be mild: it must be intravenous and can be summarized, as mentioned above for the NF, as the following options: (1) Piperacillin sodium + tazobactan sodium, + vancomycin + clindamycin. (2) Imipenem/meropenem + vancomycin + clindamycin. (3) Cefepime + vancomycin + clindamycin. As with NF, clindamycin can also be replaced by metronidazole. Such measures, evidently, must be associated with analgesia and care in an intensive care unit (ICU) setting. The prognosis is variable. In a series of 40 cases in Brazil, mortality was 22.5% and was strongly correlated with the presence of sepsis at hospital admission and the length of ICU stay; these results were very similar to those observed in a recent study from Korea with 41 patients, where the mortality rate was 22.0%.^37^, ^41^ In a wide literature review that compiled 1726 cases, the observed mortality rates ranged from 3% to 45%, with a median of 16%, and was mostly associated with sepsis and diabetes.^35^ Such data show the extreme importance of early diagnosis, specialized intervention, and support in an intensive care unit.

## Ecthyma gangrenosum

The first mention of ecthyma gangrenosum (EG) appeared in 1897 in an article by LF Barker on clinical manifestations related to an infection caused by *Baccilus pyocyaneus*, which at the time was the denomination for the current *Pseudomonas aeruginosa*.^43^ The name “ecthyma gangrenosum,” which appeared in 1951 in a publication by RH Broughton, has since been of universal use and implies, conceptually, the state of sepsis by *P. aeruginosa*.^44^ As knowledge evolved, EG became associated with other Gram-negative, Gram-positive bacteria, and even fungi, especially of the genera *Candida* and *Fusarium*.^7^, ^45^ Some authors refer to non-*P. aeruginosa* cases as ecthyma gangrenosum-like, but the main message is that the dermatological manifestation is practically identical and, for diagnostic suspicion, the first intervention should be directed to *P. aeruginosa* while the etiology of the disease is investigated.^45^, ^46^

**Figure 6:**
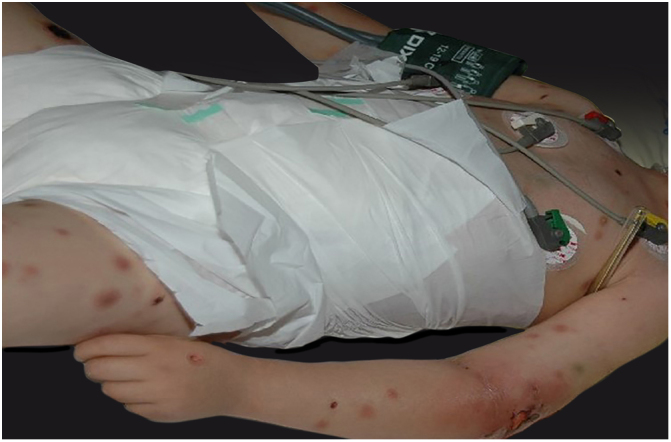
Ecthyma gangrenosum due to *Pseudomonas aeruginosa*: multiple lesions at different stages of evolution in a patient undergoing chemotherapy for myeloproliferative disease.

**Figure 7:**
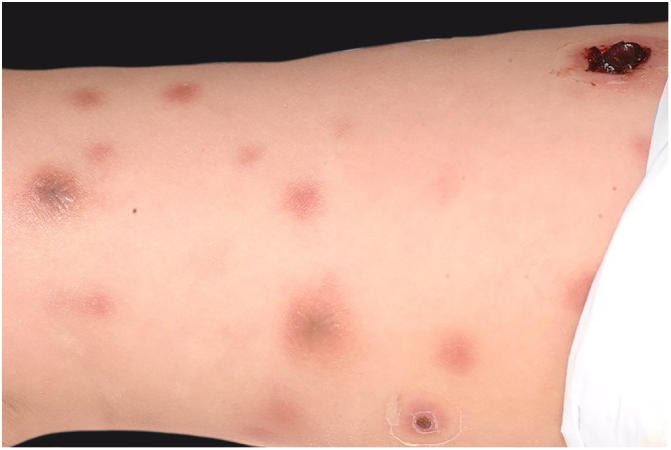
Ecthyma gangrenosum. Detail of the injury observed in Fig. 6. Presence of recent, clearly necrotic lesions.

**Figure 8:**
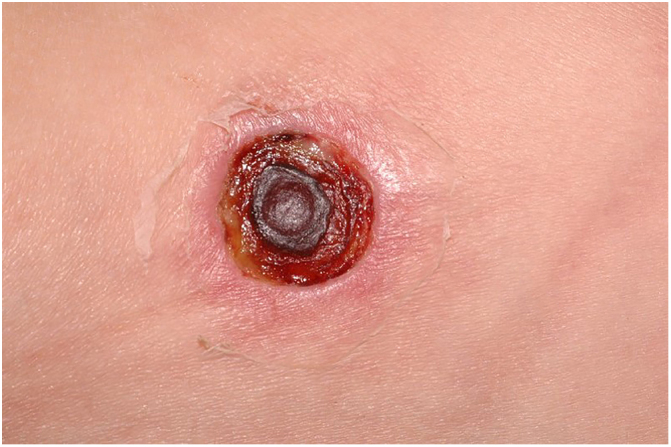
Ecthyma gangrenosum due to *Pseudomonas aeruginosa*. Detail of ulceronecrotic lesion and active border, erythematous-edematous, infiltrated.

**Figure 9:**
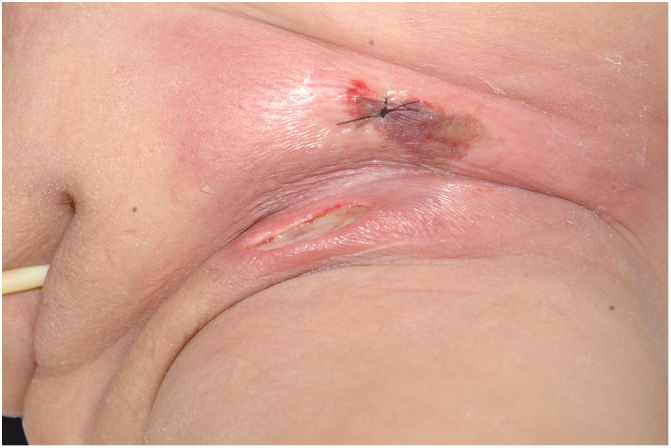
Ecthyma gangrenosum due to *Pseudomonas aeruginosa* in an infant with hitherto unknown primary neutropenia. Erythema, edema, infiltration, necrotic lesion, and recent ulcerated lesion.

Clinically, it is characterized by the rapid evolution of a localized lesion, initially vesiculobullous or papulonodular, in an erythematous, edematous background, evolving in 12–24 h to local signs of skin necrosis and an ulceronecrotic lesion.^45^, ^46^, ^47^, ^48^, ^49^ These dermatological clinical manifestations correspond to the invasion of the venules by the infectious agent, with consequent damage of the vascular wall, induction of thrombosis in the arterioles, inflammation process, edema and vascular obstruction, and localized skin necrosis. The lesions are generally small in number, but they can be multiple and at different stages of evolution (Figure 6, Figure 7, Figure 8). The preferred locations are the perineum, buttocks, inguinocrural fold (Fig. 9), intergluteal cleft, and distal extremities, but lesions can affect any area, including the head, face, and neck.^45^, ^48^, ^49^, ^50^, ^51^ The general clinical picture associated with skin lesions may already indicate fever, toxemia, or sepsis; it may even be observed in previously asymptomatic or oligosymptomatic patients.^52^, ^53^ The differential diagnosis of skin lesions should include lesions of severe small vessel leukocytoclastic vasculitis, sepsis skin lesions, disseminated vascular coagulation, and septic emboli associated with endocarditis. Although meningococcemia is mentioned in the literature as a differential of EG, purpura fulminans lesions are distinct: they are purpuric, net-like rashes without blisters.^54^, ^55^

In a review study of 167 cases of EG reported in the literature retrieved in PubMed, MEDLINE, and ScienceDirect between 1975 and 2014, the authors identified that in 73.6% of the total cases the agent was *P. aeruginosa*, in 17.3% another bacterium, and in 9% the etiology was fungal. It is noteworthy that EG was a manifestation of sepsis in only 58.5% of the cases where the agent was *P. aeruginosa*.^45^ These data are relevant, as they demonstrate that starting the empirical treatment aiming at *Pseudomonas* is the correct conduct while the real etiology is not identified, and that EG can be a manifestation of bacteremia associated with a still stable clinical picture.

EG affects any age group, but childhood as a whole deserves particular attention, as the younger the patient, the greater the severity of the prognosis.^45^, ^47^, ^49^

The predisposing factor is often immunosuppression and/or neutropenia, primary or secondary to chemotherapy. In patients with lymphoproliferative diseases undergoing chemotherapy, fungal etiology must be considered with a certain priority.^45^, ^49^, ^50^, ^51^ In general, the most prevalent underlying diseases or clinical situations are: leukemia/lymphoma, other malignancies, severe burns, transplant patients, and patients on immunosuppressive therapy.^45^, ^49^, ^50^, ^51^ In more than one case, EG was a telltale sign of severe infection by *P. aeruginosa* in the context of primary neutropenia hitherto hidden.^47^, ^49^

In the face of clinical suspicion, the etiological diagnosis relies on blood culture and culture for bacteria and fungi in a biopsy fragment of the lesion, which is collected at the edge of the ulcer or in a non-necrotic area. The antibiogram is mandatory and should not be dismissed. Simultaneously, an eventual focus for the emission of bacteremia must be identified, paying special attention to the lungs.

Treatment should immediately target the most likely etiology, *i.e.*, intravenous antibiotic coverage against *P. aeruginosa*, with the use of aminoglycosides, preferably amikacin at a dose of 7.5–15 mg/kg/day IV or IM, divided in two or three infusions/applications.^45^, ^48^ In children, the recommended dose is 15–20 mg/kg per day, with a maximum dose of 1.5 g, divided into IV infusions every eight hours or IM every 12/12 h. There is a need for dose adjustment in situations of altered creatinine clearance. The use of carbapenems is also possible. If imipenem is chosen, the recommended dose is 1–2 g/day IV infused every six hours. In children weighing less than 40 kg, the recommended dose is 15–25 mg/kg/day IV, divided into infusions every 6 h, with a maximum total dose of 2 g/day. Imipenem dose correction is required for patients with renal failure with creatinine clearance below 50 mL/min. Meropenem is the most widely used and recommended carbapenem at a dose of up to 6 g/day, IV, divided into infusions every 6 h. For children under 40 kg, the dose is limited to 20–40 mg/kg per infusion, every eight hours. In patients with renal impairment, the dose should also be corrected in light of the patient's creatinine clearance. As for possible adverse effects, the potential for nephrotoxicity and ototoxicity associated with aminoglycosides in general is noteworthy.

Surgical debridement of the lesion is a useful adjuvant method in EG, but it is not as essential as in NF and Fournier gangrene.^45^

EG prognosis depends on the patient's general condition, immunological status, underlying disease and, in cases of concomitant sepsis, on the possible delay in diagnosis before the start of treatment. In non-septic patients, EG has a lower mortality rate (16%) when compared with septic patients (38% *vs.* 96%).^56^

## Skin infections caused by methicillin-resistant *Staphylococcus aureus* (MRSA)

In the 1880s, Alexander Ogston first detected *Staphylococcus aureus* from a purulent abscess exudate located on the leg of a patient and, in 1884, Friedrich Julius Rosenbach formally isolated this bacterium.^57^ *S. aureus* is a Gram-positive bacterium coccus that is well adapted to the human host and the health care environment. It is part of the normal human microbiota, often found on the skin, especially the armpit, inguinal region, and in the nasal cavity, with a prevalence of around 25–30%. It is one of the main agents that cause endocarditis, bacteremia, pneumonia, osteomyelitis, and skin and soft tissue infections (SSTI), triggering mild to fatal conditions.^58^

*S. aureus* has quickly become one of the main causes of hospital-related infections. Initially a bacterium sensitive to penicillin, resistance was observed in the 1940s, mediated by the β-lactamase blaZ gene. In 1960, the first semi-synthetic anti-staphylococcal penicillins were developed and strains of MRSA were observed within one year of their first clinical use.^59^, ^60^

Until the 1980s, MRSA infections occurred in patients with known predisposing factors such as hospitalization, presence of an invasive device, history of surgery, hemodialysis, immunosuppression, or residence in a nursing home. Subsequently, MRSA infections were reported in healthy populations without risk factors, *i.e.*, with no recent history of contact with hospitals or health services, with an increase in the number of reports in the 2000s.^61^ The MRSA strains that caused infections in patients without the previously described risk factors were shown to be different from those in hospitals, giving rise to the term community-acquired MRSA (CA-MRSA).^62^ To differentiate hospital strains, the term hospital-acquired MRSA (HA-MRSA) was added for those related to health care services.^63^

Resistance to methicillin occurs due to chromosomal segments present in some strains of *S. aureus* that carry the methicillin resistance gene (mecA), called SCCmec (staphylococcal chromosome cassette mec), being distinct in HA-MRSA (SCCmec types I, II, and III) and CA-MRSA (SCCmec types IV–XI).^64^ MecA expression conferred resistance to the available β-lactam antibiotics, while resistance to non-β-lactam antibiotics commonly associated with HA-MRSA is due to a variety of mechanisms.^63^ The first reports of CA-MRSA in Latin America were described in 2002 and 2003 in southern Brazil.^65^

Essentially, CA-MRSA infections differ from HA-MRSA infections by three main characteristics: first, the affected populations are younger and generally healthier, with no previously defined risk factors; second, presence of epidemic clones, classified as USA300 or USA400; third, CA-MRSA clones contain a resistance mechanism produced *via* SCCmec IVa (present in 84% of CA-MARS strains) with production of Panton-Valentine leukocidin (PVL), which determines great tissue destruction, leading to severe SSTI and necrotic pneumonia.^66^, ^67^, ^68^, ^69^ It is important to highlight that the SCCmec IV pathway promotes resistance to β-lactam antibiotics in general, but not to other antibiotics, unlike HA-MRSA infections, which generally show resistance to various classes of antibiotics.^70^

Although CA-MRSA infection is not associated with the risk factors for HA-MRSA, some groups are at higher risk for developing infection by this bacterial agent, such as young adults, those incarcerated, African-Americans, illicit drug users, athletes, indigenous people, people with HIV/AIDS, and men who have sex with men.^71^

CA-MRSA is predominantly related to SSTI of varying degrees of severity; it sometimes also causes pneumonia and severe and fatal bone and joint infections.^58^, ^72^

The term SSTI is generic and can be applied to a wide variety of infections including impetigo, folliculitis, furunculosis, cellulitis, and abscesses. The focus of this review will be on cases of furunculosis and abscesses, due to the characteristic formation of purulent collections and exudation, which are often caused by MRSA.^73^

**Figure 10:**
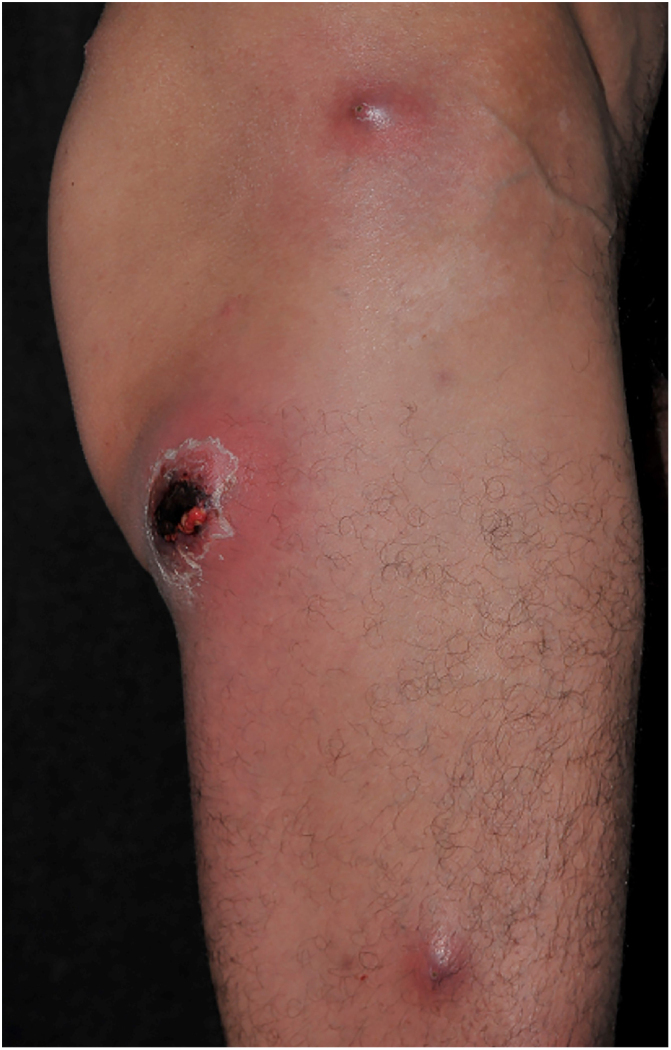
MRSA furunculosis. A healthy 42-year-old patient with several abscesses and furuncles. Positive culture for MRSA with production of Panton-Valentine leukocidin (PVL).

Furuncles mainly affect areas rich in hair follicles, such as the armpits and the gluteal region, with the formation of abscesses in the hypodermis. Hair follicles are the gateway to *S. aureus*, favoring its development. It usually presents as an erythematous, painful, and floating nodule, with pustules on the surface and a drainage point. A single or multiple concomitant lesions can be observed (Fig. 10).

**Figure 11:**
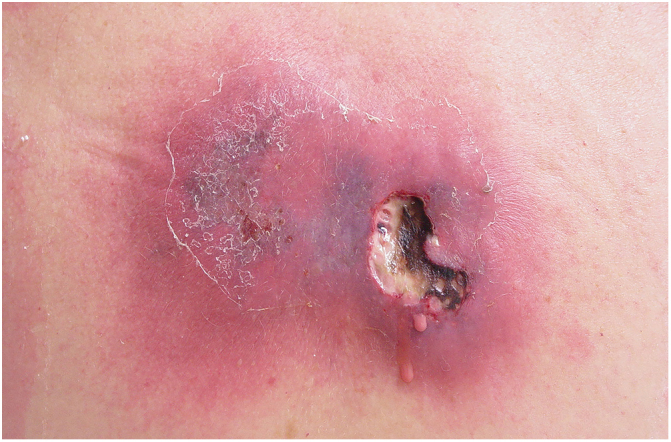
Carbuncle in a patient with insulin-dependent diabetes mellitus. An infiltrated erythematous-wine colored lesion, ulcerated with areas of necrosis and purulent secretion.

Carbuncle is the coalescence of two or more furuncles in the same locus, with multiple drainage sinus tracts, which tends to extend more deeply into the hypodermis (Fig. 11). Systemic symptoms are usually present and regional lymphadenopathy can be observed. Carbuncle can appear anywhere with hair; however, it is more common in the posterior cervical region, back, and thighs.^74^ Predisposing factors for the development of furuncles, including recurrent furuncle, are eczema, diabetes mellitus, alcohol use, malnutrition, immunodeficiency, obesity, poor hygiene, chronic MRSA colonization, hyperhidrosis, and anemia.^75^

Cutaneous abscesses are focal collections of pus located in the dermis and hypodermis, which usually present as painful, erythematous nodules, often surmounted by a pustule and with an erythematous-edematous border. They often present with floating points or signs of spontaneous drainage. In the early stages and in deeper presentations, they may not show the classic fluctuation sign. In association, cellulitis that extends radially from the purulent focus may be observed.^73^ *S. aureus* is isolated in approximately 60–75% of cases of uncomplicated skin abscesses, of which 50–70% are MRSA. Coagulase-negative staphylococcus is the next most isolated species, followed by a variety of β-hemolytic streptococcal species.^76^ Risk factors for recurrent abscesses include intramuscular injections; hair removal from legs, armpits, pubis and scalp; and previous colonization or infection with CA-MRSA.

The diagnosis of furunculosis and abscess is essentially clinical, but ultrasound can be a useful complement in cases of abscesses where fluctuation is absent or difficult to locate.^77^ When possible, sampling of purulent exudate for culture and antibiogram should be performed in order to better guide the conduct.

The treatment of isolated and small furuncles can be performed with topical antibiotics, fusidic acid, or mupirocin, three times a day, for seven to ten days. Early squeezing of the lesion should be avoided; however, surgical drainage must be performed in the fluctuation phase. As for larger furuncles, carbuncle, and abscesses, incision and drainage are strongly recommended when they are in the fluctuation phase.^78^ Incision and drainage involve a single linear incision, followed by blunt dissection. Needle aspiration has been shown to be generally inferior to incision and drainage of abscesses. However, needle aspiration may be preferred on the face, as it provides the best cosmetic results. Systemic antibiotic therapy is indicated as adjuvant, and is mandatory when there is an erythematous halo of ≥2 cm around the furuncle and in cases of carbuncle. As previously mentioned, CA-MRSA is resistant to β-lactam antibiotics, such as penicillins, first- to fourth-generation cephalosporins, carbapenems, and monobactams. Cephalosporins resistance is relevant to clinical practice, as they are among the most used antimicrobials for the treatment of SSTI and community-acquired pneumonia. Therefore, CA-MRSA infections may not be treatable by most treatment regimens empirically used for such infections.^79^ The antibiotics indicated are those with action against MRSA, such as sulfamethoxazole plus trimethoprim or clindamycin for at least seven days in uncomplicated cases, and vancomycin or daptomycin in complicated cases (*i.e.*, those with extensive involvement and toxicity or in immunocompromised patients).^78^, ^80^, ^81^

## Final remarks

Considering these data, it should be noted that the semiological training and practical and theoretical knowledge of the diseases described here and others that can be considered in the differential diagnosis make the dermatologist a key element in the suspicion, early diagnosis, and treatment/referral of potentially severe/fatal SSTI.

## Financial support

None declared.

## Authors’ contributions

Silvio Alencar Marques: Conception and planning of the study; elaboration and drafting of the manuscript, critical review of the literature; critical review of the manuscript; approval of the final version of the manuscript.

Luciana PF Abbade: Elaboration and drafting of the manuscript; critical review of the literature; critical review of the manuscript; approval of the final version of the manuscript.

## Conflicts of interest

None declared.

## Acknowledgements

To Eliete Correia Soares, photographer of the Dermatology Department at FMB-Unesp, for documenting several of the cases shown in this article. To Dr. Hamilton Ometto Stolf for the shared medical attention to the cases mentioned in this article and for his enthusiasm in the study of clinical and surgical emergencies in dermatology.

**Table:** 

**CME Questions**

1. Regarding necrotizing fasciitis (NF), check the correct alternative:

a) It is an infection that affects the superficial and even deep muscular fascia of the subcutaneous tissue, of the dermis, and of the epidermis, with evolution to local necrosis, toxemia, and possible sepsis.

b) NF that is associated with infection by multiple bacterial species (polymicrobial), called type I, is considered more frequent.

c) NF type II is the one in which Streptococcus pyogenes is the only or predominant agent and, therefore, is called monomicrobial.

d) All alternatives are correct.



2. Which of the following are correct regarding NF?

a) NF can occur even in the absence of loss of continuity of the skin.

b) The most common infectious agent is Escherichia coli.

c) Human or animal bites do not trigger NF.

d) Pain is not an important phenomenon in the natural history of NF.



3. Which of the conditions listed below constitutes a predisposing factor to NF?

a) Alcoholism and malnutrition.

b) Illicit drugs use.

c) Obesity and diabetes mellitus.

d) All of the above.



4. In the Laboratory Risk Indicator for Necrotizing Fasciitis (LRINEC) NF scoring system, the factor that most contributes to the diagnosis is:

a) Polymerase chain reaction (PCR) value.

b) Blood glucose value at admission.

c) Serum creatinine value.

d) Leukogram data on admission.



5. Considering that Fournier's gangrene is a local presentation of necrotizing fasciitis, which of the conducts listed below are correct for both diseases:

a) Broad-spectrum intravenous antibiotic therapy as soon as the diagnostic is suspected.

b) Consider the debridement of the necrotic tissue that may be present as a surgical emergency.

c) Proceed to the etiological investigation from the suspected diagnosis through blood cultures and culture of tissue fragments from the skin lesion.

d) All of the above are correct.



6. Ecthyma gangrenosum was initially described as a manifestation of bacteremia caused by:

a) Streptococcus pyogenes

b) Staphylococcus aureus

c) Escherichia coli

d) Pseudomonas aeruginosa



7. Regarding ecthyma gangrenosum, it is correct to state:

a) If carbapenems are chosen, even if there is renal failure, it is not necessary to correct the dose by creatinine clearance.

b) Treatment must be initiated upon suspicion, aimed towards infection by S. pyogenes or other Gram-positive bacteria.

c) Skin biopsy is not an adequate method to reach the etiological diagnosis.

d) Primary immunodeficiency or neutropenia are frequent predisposing factors.



8. Check the correct alternative for skin infections caused by methicillin-resistant Staphylococcus aureus (MRSA):

a) Infections caused by this agent should only be suspected in patients with a history of current or recent hospitalization.

b) Cellulitis and erysipelas without formation of purulent collections are the main skin manifestations.

c) There are two different types of MRSA strains, CA-MRSA and HA-MRSA, which occur in populations with different epidemiological profiles.

d) MRSA infections occur predominantly in immunosuppressed patients.



9. Characteristics of CA-MRSA infections are:

a) Young and healthy patients, such as athletes, and bacterial clones with production of Panton-Valentine leukocidin.

b) Patients with a history of hospitalization and resistance to β-lactam antibiotics.

c) User of illicit drugs with a history of hospitalization.

d) Immunosuppressed patients who constantly refer to health services.



10. Check the correct statement:

a) The first approach to an abscess is antibiotic therapy, while surgical drainage should be reserved for cases where there is no improvement.

b) The first choice of antibiotics for furuncles and anthrax are first-generation cephalosporins.

c) Surgical furuncle drainage is contraindicated.

d) The antibiotics of choice for treating furuncles are sulfamethoxazole plus trimethoprim or clindamycin.

**Table:** 

ANSWERS **Post-finasteride syndrome. An Bras Dermatol. 2020;95(3):271-277.**				
1. d	3. b	5. d	7. a	9. c

2. d	4. d	6. c	8. d	10.a
